# Phase-dependent modulation as a novel approach for therapeutic brain stimulation

**DOI:** 10.3389/fncom.2015.00026

**Published:** 2015-02-26

**Authors:** Ramin Azodi-Avval, Alireza Gharabaghi

**Affiliations:** ^1^Division of Functional and Restorative Neurosurgery and Division of Translational Neurosurgery, Department of Neurosurgery, Eberhard Karls UniversityTuebingen, Germany; ^2^Neuroprosthetics Research Group, Werner Reichardt Centre for Integrative Neuroscience, Eberhard Karls UniversityTuebingen, Germany

**Keywords:** brain state-dependent stimulation, closed-loop stimulation, biophysical model, coupled neuronal oscillators, phase response curve, Parkinson's disease, deep brain stimulation

## Abstract

Closed-loop paradigms provide us with the opportunity to optimize stimulation protocols for perturbation of pathological oscillatory activity in brain-related disorders. In this vein, spiking activity of motor cortex neurons and beta activity of local field potentials in the subthalamic nucleus have both been used independently of each other as neuronal signals to trigger deep brain stimulation for alleviating Parkinsonism. These approaches were superior to the standard continuous high-frequency stimulation protocols used in daily practice. However, they achieved their effects by bursts of stimulation that were applied at high-frequency as well, i.e., independent of the phase information in the stimulated region. In this context, we propose that, by timing stimulation pulses relative to the ongoing oscillation, an alternative approach, namely the targeted perturbation of pathological rhythms, could be obtained. In this modeling study, we first captured the underlying dynamics of neuronal oscillations in the human subthalamic nucleus by phased coupled neuronal oscillators. We then quantified the nature of the interaction between these coupled oscillators by obtaining a physiologically informed phase response curve from local field potentials. Reconstruction of the phase response curve predicted the sensitivity of the phase oscillator to external stimuli, revealing phase intervals that optimally maximized the degree of perturbation. We conclude that our specifically timed intervention based on the coupled oscillator concept will enable us to identify personalized ways of delivering stimulation pulses in closed-loop paradigms triggered by the phase of pathological oscillations. This will pave the way for novel physiological insights and substantial clinical benefits. In addition, this precisely phased modulation may be capable of modifying the effective interactions between oscillators in an entirely new manner.

## Introduction

Brain neuromodulation by deep brain stimulation (DBS) is meanwhile a recognized form of treatment for several neurological and neuropsychiatric disorders such as severe Parkinson's disease (PD) (Schuepbach et al., 2013), dystonia (Vidailhet et al., 2005), and essential tremor (Deuschl et al., 2011). However, general application of this therapeutic modality remains limited. This might be due to stimulation-induced side effects and/or partial efficacy of the intervention which is probably related to a misalignment between stimulation parameters and the current disease state (Moro et al., 2006; Mure et al., 2011). While patients often display variable clinical symptoms, the stimulation parameters, such as continuous high-frequency stimulation, are predefined and remain unchanged until manual modifications are performed by the physician in charge.

Closed-loop paradigms modulating the stimulation parameters on the basis of online recorded physiological markers provide us with the opportunity to adjust stimulation protocols and improve therapeutic efficacy. In this regard, the first studies in both non-human primates (Rosin et al., 2011) and Parkinsonian patients (Little et al., 2013) addressed the current limitations by applying stimulation in an adaptive manner only when specific physiological markers were detected. More specifically, adaptive DBS of the globus pallidus internus controlled by spiking activity of motor cortex neurons was more effective than continuous high-frequency stimulation in a non-human primate model of PD (Rosin et al., 2011). In a recent clinical study, adaptive DBS of the subthalamic nucleus (STN)—triggered by beta-band activity (a physiological marker of motor impairment in PD) and recorded in the immediate vicinity of the stimulating electrode in the STN—was shown to be more energy-efficient than, and clinically superior to continuous high-frequency DBS (Little et al., 2013). Despite being superior to the standard stimulation protocols, these closed-loop approaches nonetheless achieved their effects by bursts of stimulation applied at high frequency independent of the phase information in the stimulated region (Rosin et al., 2011; Little et al., 2013).

In this context, we propose that, by timing stimulation pulses relative to the ongoing oscillation in the respective area, an alternative approach—namely the targeted perturbation of pathological rhythms—can be introduced. We therefore performed a modeling study to determine the essential dynamics of neuronal oscillations in the human subthalamic nucleus by phased coupled neuronal oscillators in an aim to identify those phase intervals in which stimulation would maximize the degree of perturbation of pathological rhythms.

## Materials and methods

The present modeling study is based on intraoperative electrophysiological recordings of two PD patients who underwent standard DBS surgery with bilateral electrode implantation in the STN and was performed in accordance with the guidelines of the local ethics committee of the Medical Faculty of the University of Tuebingen. This data represents spontaneous brain activity recorded for about 3 min through the final quadripolar DBS electrode (model 3389, Medtronic, Inc., Minneapolis, MN) which was implanted in one brain hemisphere while electrode insertion was prepared for the second side. The STN was localized via direct targeting on preoperative magnetic resonance imaging (Foltynie et al., 2011) and then during surgery with online electrophysiology (Chen et al., 2006; Holdefer et al., 2010). In accordance with standard operating procedures, dopamine medication was not administered for the last time 12 or more hours prior to surgery to avoid interference with intraoperative recordings and clinical testing (Hammond et al., 2007). In addition, intraoperative propofol medication was discontinued about 30 min before electrophysiological recordings were initiated (Raz et al., 2010). Local field potential (LFP) signals were continuously sampled at 1.4 KHz and amplified by factor 50. The reference electrodes were attached to the ears and the ground was placed on the nasion. The impedance for the intracranial electrode was ~1KΩ. Stereotactic planning (Machado et al., 2006) was based on multimodal preoperative images (1 mm slice thickness) from contrast-enhanced magnetic resonance imaging (MRI) and computer tomography (CT) imaging (Siemens, Erlangen, Germany). Standard electrophysiological recordings (AlphaOmega, Nazareth, Israel) and clinical test stimulation were performed intraoperatively to adjust electrode localization. This was later confirmed by postoperative MR and CT imaging.

### Phase synchronization

**Figure 1:**
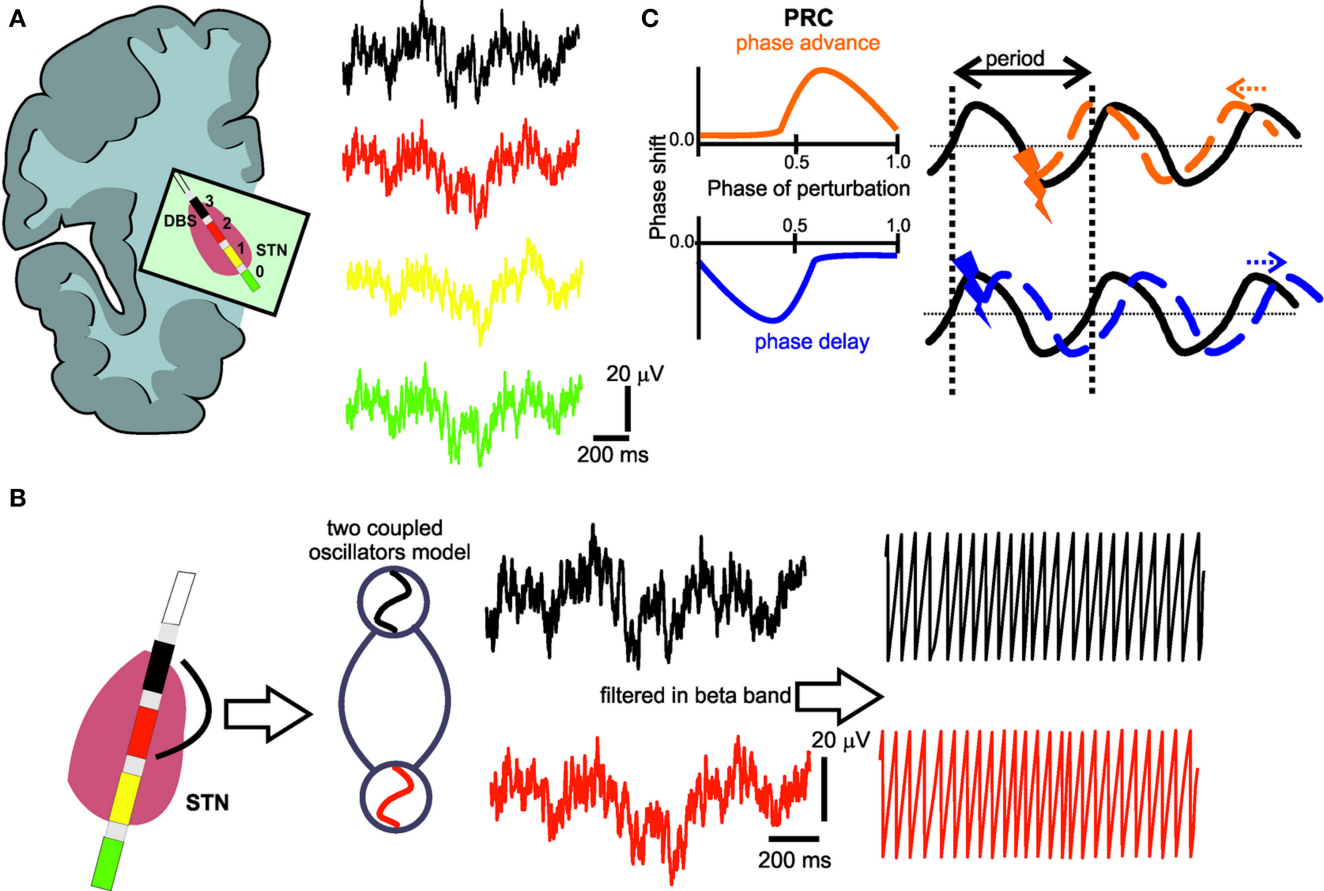
**(A)** Deep brain stimulation lead positioned in the subthalamic nucleus region with four electrode contacts (0–3) and respective local field potential (LFP) recordings. **(B)** The LFPs from the electrode contact (black) with the most pronounced oscillatory activity in the beta-band and the electrode contact (red) with the most prominent phase synchronization to the first contact are captured as coupled neural oscillators. In a second step, the phase of these oscillators is extracted by Hilbert transform. **(C)** Illustration of the typical characteristics of a phase response curve (PRC) with positive (orange) or negative (blue) values indicating an earlier or later start of the perturbated cycle, respectively, i.e., the perturbation could cause a positive (orange) phase shift (phase advance), or a negative (blue) phase shift (phase delay), depending on the timing of the perturbating stimulus. The black curve represents the ongoing oscillation in the absence of any perturbation.

The implanted quadripolar electrode (Figure 1A) enabled us to independently record four oscillatory sources, i.e., local field potentials (LFP), in the target region and to quantify their neural interaction. For this purpose, we used the phase approach proposed by Rosenblum and Pikovsky (2001) to reconstruct the phase response curve (PRC) of the LFP signals recorded in the STN. The concept of phase synchronization between coupled oscillators has not been used to derive a phase-response curve of brain signals yet, but has already been applied in several electrophysiological studies in different functional systems (Kralemann et al., 2013; Zhu et al., 2013). However, brain signals are particularly suited for such an approach as filtered LFP signals at a given frequency band have a sinusoidal waveform similar to an oscillator. Therefore, phase modeling might capture the neural network dynamics of coupled oscillators (Wang, 2010).

**Figure 2:**
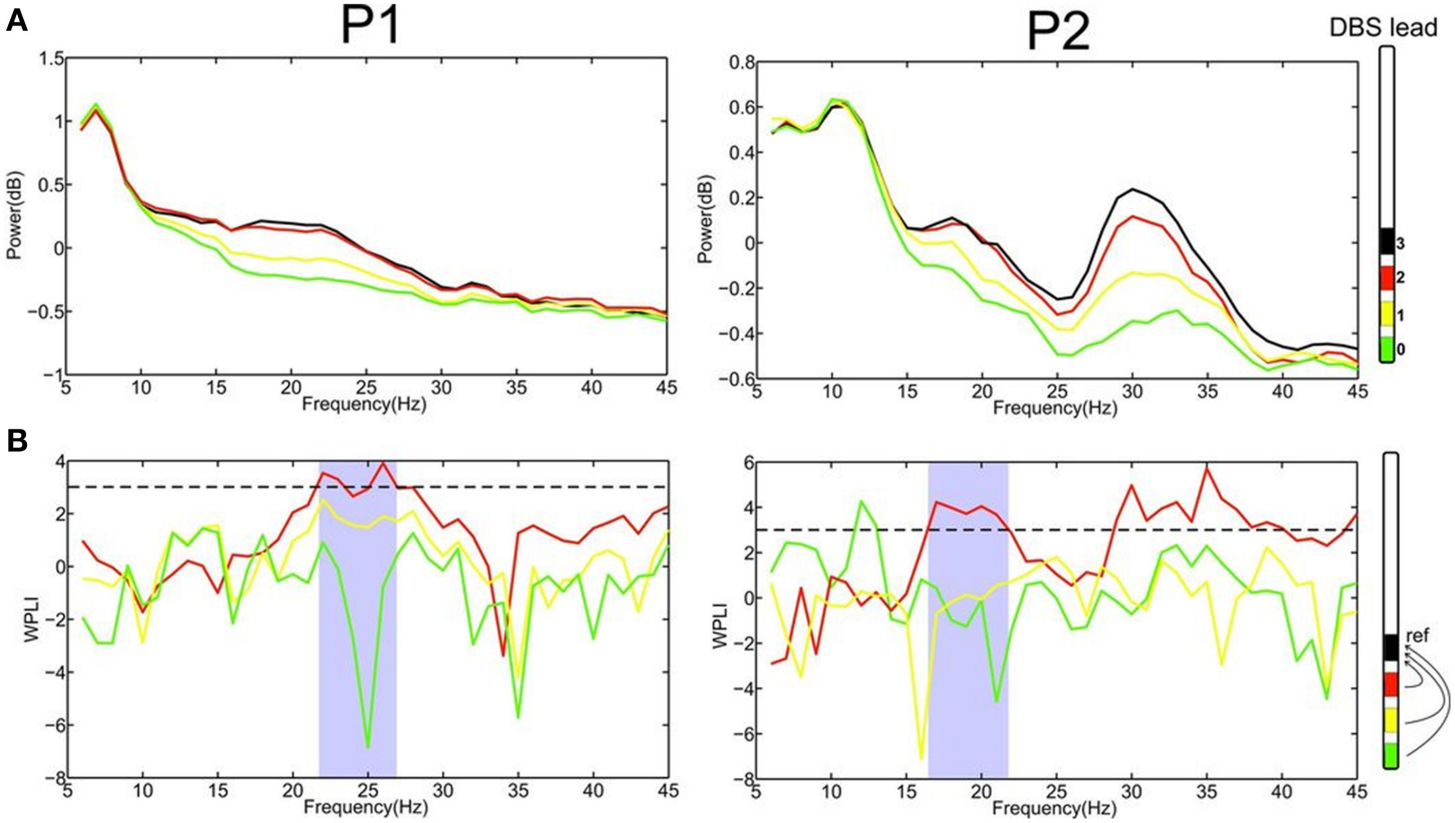
**(A)** Power spectrum of LFPs recordings at the four different contacts (0–3) for patient 1 and 2, respectively, revealed that the most upper electrode contact (3, black) of the quadripolar lead has the highest spectral power in the beta-frequency band. **(B)** Computing phase synchronization between electrode contact 3 (black) and the others applying the Weighted Phase Lag Index (WPLI) showed that the most prominent coupling is with contact 2 (red). The statistically significant frequency range for WPLI is indicated in gray. The dashed line represents the significance threshold (3, corresponding *p* < 0.003).

Data analysis of LFPs were performed in Matlab (The Mathworks, Natick, Massachusetts, USA) using custom-made scripts, DAMOCO toolbox (Kralemann et al., 2007, 2008) and Fieldtrip, an open source analysis toolbox (Oostenveld et al., 2011). To reconstruct the PRC, the power of LFP was first computed by the multitaper method. Maximum spectral estimation was provided by a single Hanning taper (Mitra and Pesaran, 1999). This approach enabled us to identify the specific electrode contact that displayed pronounced oscillatory activity in the beta-band—a known pathophysiological marker of motor impairment in PD (Little and Brown, 2012)—and which we used as a reference for the further computing. We next applied the Weighted Phase Lag Index (WPLI) (Vinck et al., 2011) to detect the electrode contact displaying the most prominent phase synchronization with this reference electrode. WPLI is defined as follows:

(1) $$\begin{array}{l} WPLI_{LFP-LFP}\\ \;=\frac{n^{-1}\sum_{n\;=\;1}^{N}\left|imag\left(S_{LFP\;-\;LFP\;n}\right)\right|sgn\left(imag\left(S_{LFP\;-\;LFP\;n}\right)\right)}{n^{-1}\sum_{n\;=\;1}^{N}imag\left(S_{LFP\;-\;LFPn}\right)} \end{array}$$

where *S* is the cross-spectrum density matrix, *imag* the imaginary part and *sgn* the sign function. Moreover, WPLI is insensitive to volume conduction effects since it eliminates zero phase lag signals. Thereby, it is more sensitive to detect true phase interaction as compared to common phase measurements such as the imaginary part of coherence (Nolte et al., 2004). The value of WPLI was standardized by an estimate of its standard deviation and values beyond threshold of 3 (corresponding *p* < 0.003) were considered statistically significant (Nolte et al., 2004; Hohlefeld et al., 2013, 2014). This statistically significant frequency range (see Figure 2B) was used for further analysis, i.e., phase extraction.

### Physiologically informed phase response curve

Having determined the significant frequency interval of neural interaction for each patient, we went on to filter the LFP in that particular, i.e., patient-specific, frequency range using a Kaiser FIR filter with the MATLAB filtfilt function to avoid phase distortions. The unwrapped phase of each sample of the filtered LFP was then computed using the Hilbert transform (Figure 1B). The method proposed by Rosenblum and collaborators (Kralemann et al., 2007, 2008) was then used to empirically reconstruct the phase coupling function between the two electrode contacts in the STN that had already been determined. This method adapts the empirical phases to a generic model of two coupled oscillators as follows:

(2) $$\begin{array}{l} {\dot{\varphi }}_{1}=\omega_{1}+F_{2\to 1}(\varphi_{1},\varphi_{2})\\ {\dot{\varphi }}_{2}=\omega_{2}+F_{1\to 2}(\varphi_{2},\varphi_{1}) \end{array}$$

where the dot represents the derivative, while φ and ω are the phase and autonomous frequency, respectively. Since the extracted phase is non-universe θ_1,2_, a transformation is needed to provide an invariant description of the coupled dynamics. The transformation from the unwrapped phase to the genuine phase of *N* observations is defined as following,

(3) $$\varphi =\theta +2\pi \sum_{n\;\neq \;1}\frac{S_{n}}{in}\left(e^{in\theta }-1\right)$$

where

$$S_{n}=\frac{1}{N}\sum_{j\;=\;1}^{N}e^{in\theta \left(j\right)}.$$

After this phase correction is performed, the coupling function *F* of two coupled systems can be approximated by the Fourier series:

(4) $$F=\sum_{n,m}A_{n,m}e^{-i\left(n\varphi_{2}\;+\;m\varphi_{1}\right)}$$

with coefficients

$$A_{n,m}=\int_{0}^{2\pi }\int \varphi_{1}e^{-in\varphi_{1}\;-\;im\varphi_{2}}$$

where *n* and *m* are indices synonymous to the *n*:*m* phase locking the indices of two oscillators and *A* refers to the coefficients of the respective Fourier series (Kralemann et al., 2007, 2008). The resulting PRC reflects the interaction of the oscillators. When the PRC results in positive or negative values (Smeal et al., 2010), it indicates the cycle to start sooner or later, respectively, i.e., a perturbation could cause a positive phase shift (phase advance), or a negative phase shift (phase delay) (Figure 1C).

## Results

**Figure 3:**
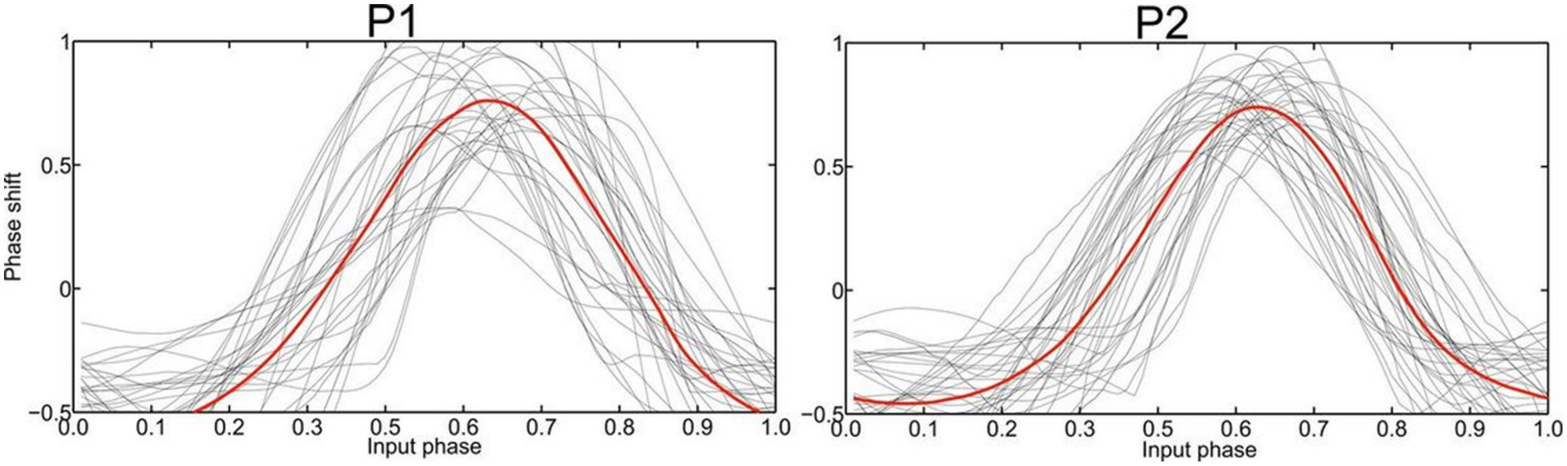
**Empirically determined PRC phase response curves (PRC) of patient 1 and 2, respectively, with the averaged curve indicated in red**. The strongest phase-coupling effect in both P1 and P2 was observed around φ = 0.6 with the highest positive value, i.e., the maximum phase advance.

The recording of local field potentials (LFP) revealed that the most upper electrode contact of the quadripolar lead has the highest spectral power in the beta-frequency band (15–30 Hz, Figure 2A), indicating that it is located in the sensorimotor part of the STN (Figure 2A) (Holdefer et al., 2010; Yoshida et al., 2010; Zaidel et al., 2010; Novak et al., 2011; Deffains et al., 2014). Using this electrode contact as a reference, the neighboring contact revealed the most prominent phase synchronization when applying the Weighted Phase Lag Index (WPLI). This phase coherence showed a significant synchronization in the frequency range between 23–27 and 17–22 Hz for P1 and P2, respectively (Figure 2B), indicating that there is a pathological increase of functional connectivity in the sensorimotor part of the STN (Pogosyan et al., 2010). By applying a modeling approach of two neural oscillators characterized by phase dependency, we succeeded in quantifying this interaction by a phase response curve. The inherent variability of the PCRs resulting from the empirical data was resolved using the similarity method (Kralemann et al., 2013). PRC extracted from both P1 and P2 showed a similar pattern with two different domains (Figure 3): a phase delay in the 0 < φ < ~0.3 and ~0.7 < φ < 1.0 intervals and a strong phase advance in the ~0.3 < φ < 0.7 interval. The strongest phase-coupling effect was observed around φ = 0.6.

## Discussion

The beta-band functional connectivity observed within the STN tallies well-with earlier work, indicating that this synchronization reflects a pathological marker for different motor features of PD (Pogosyan et al., 2010; Hohlefeld et al., 2013). We also located the most prominent phase synchronization in the dorsal, sensorimotor part of the STN (Pogosyan et al., 2010), i.e., the area known to be clinically most effective in suppressing PD symptoms during DBS (Herzog et al., 2004; Yokoyama et al., 2006; Schlaier et al., 2014). It remains to be experimentally disentangled, whether additional physiological markers may capture a broader spectrum of PD symptoms and may therefore be better suited for closed-loop applications. However, more sophisticated approaches of simultaneous sensing and stimulation with online signal processing may decrease the battery life span and should therefore be balanced with potential clinical benefits (Little and Brown, 2014).

**Figure 4:**
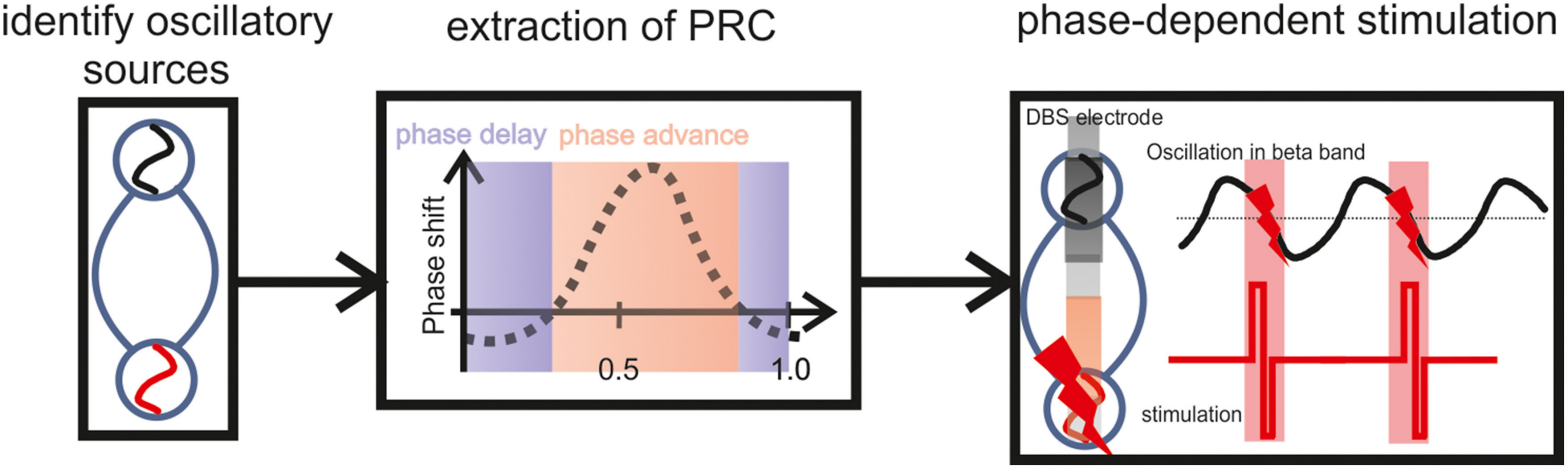
**Illustration of a closed-loop modulation protocol, recording and stimulating with two adjacent DBS electrode contacts in the STN, triggered by the ongoing oscillatory phase estimated on the basis of the PRC curve**. To determine this precise timing, a closed-loop system will require a physiological calibration for each patient including the estimation of WPLI and PRC based on the individual *vivo* recordings. After this calibration, i.e., based on the subject-specific frequency range and the precise phase of maximum perturbation, the respective phase information will be the feedback signal to control the stimulation.

On the basis of these findings, phase-specific interventions appear to be the most straightforward approach for specifically perturbating pathological synchronization. The PRC disentangles the timing of the regional interaction in the dorsal STN to reveal the highest synchronization between the two oscillators at phase 0.6 (Figure 4). In fact, the PRC also reveals phase sensitivity of an oscillator to external perturbation (Smeal et al., 2010). We therefore propose that the application of DBS stimuli at this phase, i.e., replacing one of the two oscillators by an external stimulus, maximizes perturbation of the pathological state. Along these lines, the effect of a perturbation on an oscillating system is known to depend on the phase at which the perturbation is applied (Pikovsky et al., 2001; Kralemann et al., 2013). This assumption is further supported by physiologically calibrated modeling results which suggest that precisely timed stimulation pulses could indeed be used to shift the phase of oscillations (Witt et al., 2013).

Moreover, when stimuli are properly phased with respect to ongoing oscillations, they might even induce long-term potentiation/depotentiation (LTP/LTD) effects (Martin et al., 2000; Kauer and Malenka, 2007): both *in vitro* (Huerta and Lisman, 1993) and *in vivo* (Pavlides et al., 1988) experimental studies reported LTD induction when stimuli were applied during the positive phase of the theta rhythm. Similarly, depotentiation of existing LTP was achieved when stimuli were phase-locked to the negative phase of theta (Huerta and Lisman, 1995). Phase-specific pulses may therefore induce bidirectional modifications of synaptic strength (Martin et al., 2000) with the potential to turn a whole network into a synchronized or a desynchronized state (Pfister and Tass, 2010).

Future closed-loop modulation protocols will require simultaneous recording and stimulation. The presented findings suggest that two adjacent DBS electrode contacts in the STN may be used for this purpose, i.e., to record and to stimulate, respectively. In such a scenario the stimulating contact would be triggered by the ongoing oscillatory phase recorded at the adjacent electrode and estimated on the basis of the PRC curve. To determine the precise timing, a physiological calibration of the closed-loop system will be required for each patient beforehand, including the estimation of WPLI and PRC based on the individual *vivo* recordings. After this calibration, i.e., based on the subject-specific frequency range and the precise phase of maximum perturbation, the respective phase information will be the feedback signal to control the stimulation. This approach can be implemented with online algorithms, i.e., real-time calculation of the instantaneous phase based on the Hilbert transform (Figure 4).

Phase-dependent stimulation protocols may therefore be capable of modifying the effective interactions between oscillators in an entirely new fashion, potentially inducing lasting effects mediated by LTP/LTD. Furthermore, with regard to different neurological and neuropsychiatric disorders, such an approach would transform brain stimulation from a symptomatic intervention and temporary modulation—displaying its effects only for the duration of stimulation—to a treatment option that induces long-term plastic changes and durable effects lasting beyond stimulation.

### Conflict of interest statement

The authors declare that the research was conducted in the absence of any commercial or financial relationships that could be construed as a potential conflict of interest.
